# Development and Validation of a GC/MS Method for Simultaneous Determination of 7 Monoterpens in Two Commercial Pharmaceutical Dosage Forms

**Published:** 2018

**Authors:** Marjan Esfahanizadeh, Seyed Abdolmajid Ayatollahi, Ali Goodarzi, Mitra Bayat, Athar Ata, Farzad Kobarfard

**Affiliations:** a *Phytochemistry Research Center, Shahid Beheshti University of Medical Sciences, Tehran, Iran. *; b *Department of Medicinal Chemistry, School of Pharmacy, Shahid Beheshti University of Medical Sciences, Tehran, Iran.*; c *Department of Chemistry, Richardson College of the Environmental Science Complex, The University of Winnipeg, Winnipeg, Canada.*; d *Incubation Center for Pharmaceutical Technologies, Shahid Beheshti University of Medical Sciences, Tehran, Iran.*

**Keywords:** Monoterpenes, GC-Mass, Validation, Determination, Rowachol, Rowatinex

## Abstract

Terpenes are active constituents of many pharmaceutical dosage forms with natural products origin. One of the challenges in developing dosage forms with herbal origin is their standardization as pharmaceutical products. GC-Mass is the most decisive and reliable technique to fulfill the requirements in this regard. In the present study, a reliable, rapid, and accurate method was developed for determination of 7 monoterpenoids in two selected pharmaceutical dosage forms (rowatinex and rowachol soft gelatin capsules) by gas chromatography-mass spectrometry triple quadrupole selected ion monitoring GC/MS-TQ-SIM. The method was validated for various parameters such as precision, linearity, accuracy, solution stability, limit of detection, and quantification. The average recovery of terpens was in the range of 91.6-105.7%. The method was proved to be repeatable with RSD in the range of 0.28-11.18 for all of the concentration levels. The developed method is simple, rapid, and sensitive and was applied for determination of alpha pinene, camphene, beta pinene, cineol, fenchone, borneol, trans-anethol and menthol in a few batches of rowachol and rowatinex capsules purchased from local drug stores.

## Introduction

Monoterpenes are widely abundant volatile compounds which are distributed in the plant kingdom and are responsible for the organoleptic properties associated with various herbs, spices, citrus, conifers and most flowers and fruits. They are chemically 10-carbon, short chain, and volatile compounds which are mostly found in essential oils extracted from medicinal plants. 

Monoterpenes have also been used in a wide variety of pharmaceutical products as both active ingredients and excipients. They have antiseptic, antiviral, and bactericidal effects (Table 1) (1-8).

In the field of pharmaceutical formulation, it is believed that the therapeutic effect of the drugs could be enhanced by combination of natural compounds with chemical (synthetic) drugs. Dias *et al.* have reported a formulation in which sage essential oil (SEO) bicyclic monoterpenes with antiflogistic, antiseptic and antimycotic properties were combined with terbinafine (TB) having a strong antimycotic activity (9).

Aromatherapists use monoterpenes as a mucus membrane tonic as decongestants and to ease nasal and other mucus membrane discomforts. Monoterpenes evaporate easily and have a low boiling point and they are mostly colorless and prone to oxidation. 

Monoterpenes have also been used as drug shuttles for cisplatin derivatives to overcome the resistance of cancer cells to this drug (11).

Beside other effects, terpenes are known to have diuretic and antibacterial effects as well as spasmolytic and hyperemic effects. Consequently, terpenes may have the potential for use in the medical treatment of urinary tract pathologies, such as stone disease (3, 9 and 10).

Because of the pharmaceutical importance of monoterpenes there is a need for a reliable validated method for their determination in pharmaceutical dosage forms.

In most cases, monoterpenes lack a UV chromophor group in their structures which makes them transparent and not detectable by commonly used UV detectors.

The volatile nature of monoterpenes makes them amenable to gas chromatographic system and the incomparable capabilities of mass detector could be exploited coupled to the GC system to develop a sensitive and robust method for determination of monoterpenes. In the present work two pharmaceutical dosage forms were selected in which a combination of several monoterpenes have been used as active ingredients and a GC-Mass method was developed for determination of all monoterpenes in these medications. The two products are Rowatinex and Rowachol (13). Among the seven active ingredients of rowatinex, pinene, camphene, cineol, fenchone, and borneol are monoterpenes and only trans-anethol is a phenylpropane derivative.

Rowatinex and Rowachol (ROWA Pharmaceuticals Ltd., Bantry, Co. Cork, Ireland) are two medical products which are used in treatment of bile stone and urolithiasis respectively. These two products contain a combination of seven naturally occurring small molecules (mostly terpenes) which are categorized as natural products and therefore they are considered as medications with herbal origin (Table 2) (1, 3). Challenge of herbal medicines is their standardization. The present study was a primary attempt to develop an in house assay method for standardization of Rowatinex and Rowachol. The results could be used in postmarketing survey (PMS) studies.

## Experimental

*Materials and Methods*

All monoterpens standards were purchased from Merck (Germany). All organic solvents were at least of LC grade and purchased from Merck (Germany).

*GC-TQ/MS*

Analyses were carried out by using a 7000 Agilent triple Quadrupole MS system coupled with a 7890A GC, equipped with a split/splitless injection port, an autosampler model Agilent 7693, and electronic ionization. A HP-5MS 5% Phenyl Methyl Silox, Agilent 19091s-433 capillary column was used (30 m × 0.25 mm I.D. and 0.25 μm film thickness). Helium with a purity of 99.99% and a flow rate of 1 mLmin^-1^ was used as carrier gas. The samples were injected into the GC-Mass system in a split injection mode (Split ratio 1:10).

The temperature of injection port, the ion source, the quadropole and transfer line were set at 300, 230, 100, and 300 °C, respectively. For the identification of the analytes, present in the samples the mass spectrometer was operated in full scan mode. For quantification and validation, the mass spectrometer was operated in SIM mode (100 msec dwell time). For quantification a calibration line was constructed and calculated using ordinary least squares regression.

**Table 1:** Structure and pharmacological effects of monoterpenes

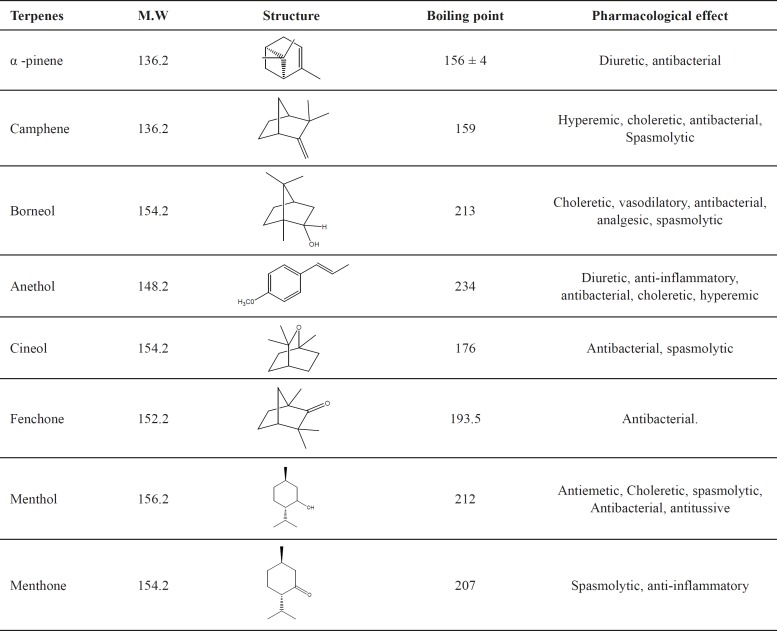

**Table 2:** Composition of monoterpens in Rowatinex (%).

**pinene**	**Camphene**	**Cineol**	**Fenchone**	**Borneol**	**Trans-anethol**	**Olive oil**
31	15	3	4	10	4	33
**Composition of monoterpens in Rowachol (%)**						
**pinene**	**Camphene**	**Cineol**	**Menthol**	**Borneol**	**Menthone**	**Olive oil**
17	15	2	32	5	6	33

**Table 3:** Concentrations of monoterpens in mixed stock standard solution for rowatinex

**Concentrations (mg/100 mg)**						
**α -pinene**	**β -pinene**	**Camphene**	**Cineol**	**Fenchone**	**Borneol**	**Trans-anethol**
33	8.24	20	4	5.3	13.3	5.3
**Concentrations of monoterpens in mixed stock standard solution for rowachol**						
**Concentrations (mg/100 mg)**						
**α -pinene**	**β -pinene**	**Camphene**	**Cineol**	**Menthol**	**Borneol**	**Menthone**
33	8.24	6.64	2.65	42.5	6.64	8

**Table 4:** Rowatinex and Rowachol time segments

Rowatinex time segments			Rowatinex time segments	
**Time segment (min)**	**Monitored ion**		**Time segment (min)**	**Monitored ion**
6-7.3	106		6-7	106
7.3-8.8	136, 121, 93		7-8.8	136, 121, 93
8.8-9.7	121, 154			
9.7-10.5	81, 69		8.8-9.7	121, 154
10.5-11.8	139, 154		9.7-14	121
11.8-25	147, 117		14-25	112, 139, 154, 95,123,128

**Table 5:** The retention times, diagnostic ions and selected quantification ion for the target monoterpens and internal standard

**No.**	**Compound**	**Retention time**	**Diagnostic ions**	**Quantification ion**
1	p- Xylene	6.9 a, b*	106, 91, 77	106
2	α- pinene	7.4^a^ 7.5^b^	136, 121, 93	136
3	Camphene	7.6^a^,^ b^	136, 121, 93	121
4	β- pinene	8.13^a^ 8.3^b^	136, 121, 93	136
5	Cineol	9.01 a, b	154, 139, 108	154
6	Fenchone	9.9^a^	69, 81, 152	81
7	Borneol	10.9^a^- 15.5^b^	95, 110, 139	139
8	Trans-anethol	12.06^a^	117, 147, 148	117
9	Menthol	15.9^b^	95, 123, 138	138
10	Menthone	14.3^b^	112, 139, 154	112

a Temperature program a.

b Temperature program b.

**Table 6:** Calibration data (equation and regression coefficient) of 9 monoterpens in spiked samples calibration curves

**No.**	Compound	**Equation**	**Regression coefficient**
1	α- pinene	y = 0.0126x - 0.0046	0.9991
2	Camphene	y = 0.0181x - 0.0033	0.9984
3	β- pinene	y = 0.0157x - 0.0016	0.9981
4	Cineol	y = 0.0414x - 0.0024	0.9987
5	Fenchone	y = 0.1575x - 0.0479	0.9972
6	Borneol	y = 0.0121x - 0.0047	0.9986
7	Trans-anethol	y = 0.0293x - 0.0044	0.9996
8	Menthol	y = 0.014x - 0.0027	0.9946
9	Menthone	y = 0.0559x - 0.0242	0.9988

**Table 7:** Average recoveries (%) and range of relative standard deviations (%) obtained by GC-MS analysis of monoterpenes in Rowatinex and Rowachol capsules

**Compound**	**Average recovery (%) n = 3**			**Total Recovery (%) n = 9**	**Range of RSDs (%)**
	**0.5**	**0.25**	**0.125**		
α- pinene	101.2	94.4	100.73	98.7	1.5- 5.5
β –pinene	95.6	92.5	86.9	91.6	5-15
Camphene	103.7	96.6	97.3	99.2	1-4.5
Cineol	97.4	92.6	87.2	92.4	4-13
Fenchone	102.7	100.8	86.1	96.5	5-15
Borneol	101.3	99.8	106.9	102.6	2-8
Trans-anethol	101.4	98.9	104.2	101.5	2-5
Menthone	102.6	97.2	104.3	101.36	2-6
Menthol	97.96	109.2	115	109.03	4-15

**Figure 1:**
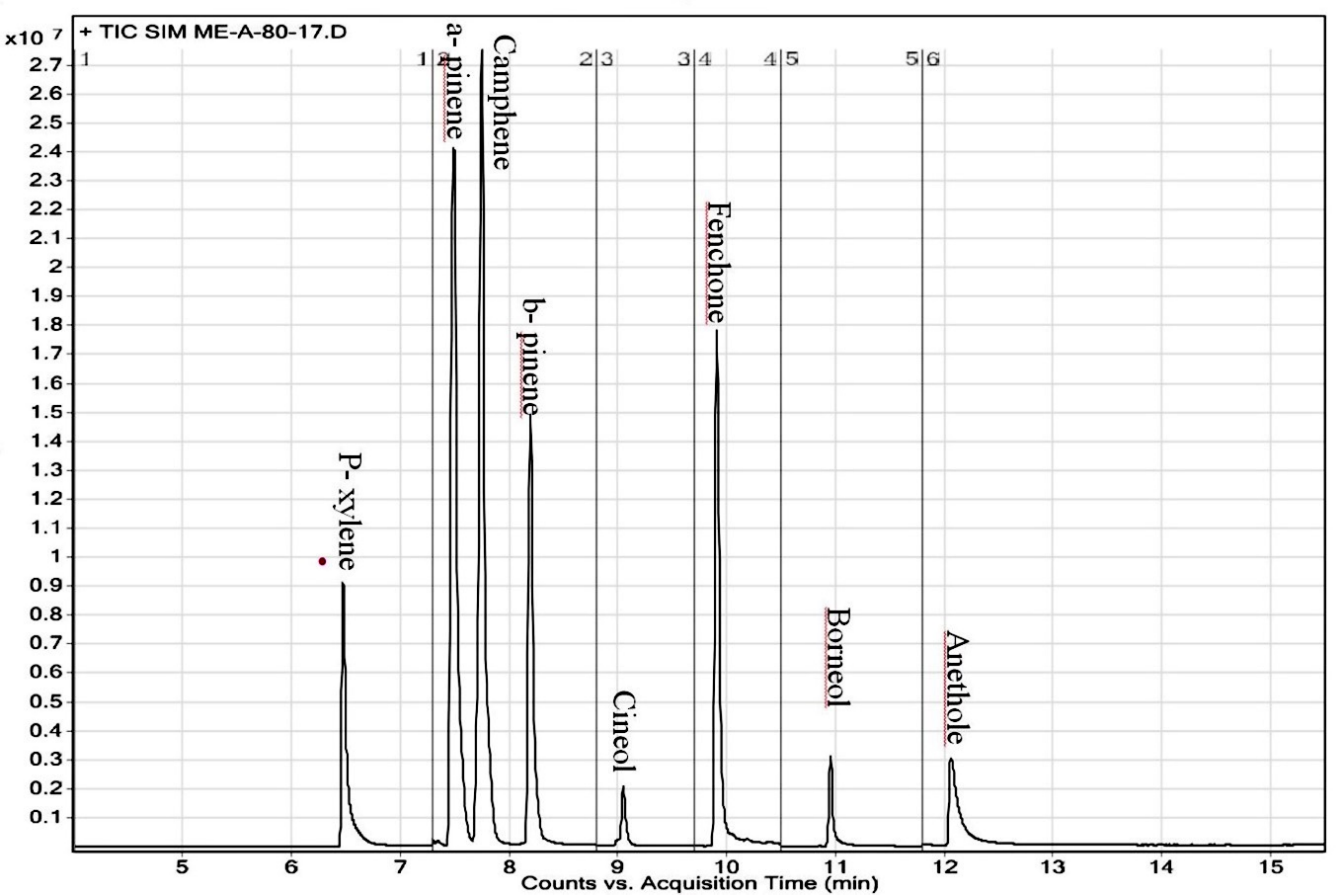
Representative TIC-SIM chromatogram for Rowatinex constituents

**Figure 2:**
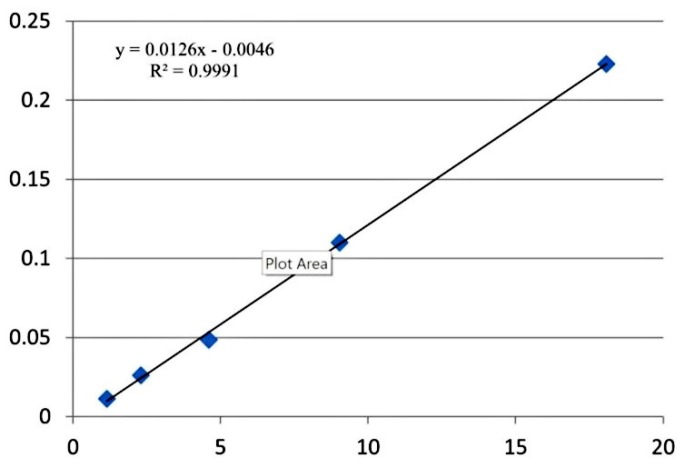
Calibration curve for α –pinene in mixed sample

*Calibration standards*

A mixed standard stock solution was prepared in olive oil specified in Table 3. The standards for the calibration curve were prepared in volumetric vials using mixed standard stock solution by serial dilution to yield 1/2, 1/4, 1/8, and 1/16 of the original concentration of each monoterpen in stock solution. In order to inject the standard solutions, 10 μL of each concentration was added to 990 μL chloroform followed by addition of 1 μL of internal standard solution (10 mg/mL of para-xylene in chloroform). The selection of the internal standard was based on an initial screening of a few different organic compounds considering their retention time and peak shape. 1 μL of samples were injected into gas chromatograph.

*Quality control preparation*

A second mixed standard solution independent of the mixed stock solutions was used for the preparation of the quality control samples (QCs). QC samples were prepared in volumetric vials using QC stock solutions by serial dilution to yield 1/2, 1/4, and 1/8 of the original concentration of each monoterpen.

*Assay preparation*

The content of ten soft gelatin capsules of each Rowatinex and Rowachol from the same batch was mixed separately and 10 μL of the mixture was added to 990 μL chloroform followed by addition of 1 μL internal standard solution and 1 μL of this solution was injected into GC-MS system.

The process was repeated for three different batches of Rowatinex and Rowachol. The concentration of each monoterpene was determined by interpolation of its area ratio of that monoterpene on its calibration curve. All determinations were conducted in triplicates.

*GC/TQ/MS*

The GC/TQ/MS was employed with helium as the carrier gas at the constant flow of 1 mL/min. After acquisition of the total ion chromatogram for the mixed stock standard solutions in scan mode, the peaks were identified by their retention time and mass spectra. The identification was confirmed by comparing the relative abundances for three ions (one quantifier and two qualifiers) of the experimental standards to know relative abundances of the NIST MS Library reference spectra. The most abundant ion that showed no evidence of chromatographic interference and had the highest signal to noise ratio was taken for quantification purposes. A representative GC-TQ-MS chromatogram of 7 monoterpens and internal standard (p- xylene) is shown in Figure 1.

The temperature program used for separation of Rowatinex constituents was as follow: 

The oven temperature started at 50 °C and remained at this temperature for 2 min, increasing to 116 °C at 10 °C/min ramp rate, increasing to 143 °C at 15 °C/min and then going to 220 °C at 30 °C/min followed by increasing the temperature to 290 °C at 60 °C/min and remaining at 290 °C for 8 min (Temperature program a).

Selected ion monitoring (SIM) was used for quantification of each analyte. Table 4 shows the m/z used for each analyte and the time segment during which, each ion is monitored. Dwell time was adjusted at 100 msec.

The temperature program used for separation of Rowachol constituents was as follows: 

The oven temperature started at 50 °C and remained at this temperature for 2 min, increasing to 130 °C at 10 °C/min ramp rate, decreasing to 70 °C at 50 °C/min ramp rate, finally increasing to 290 °C at 30 °C/min and remaining at this temperature for 10 min (Temperature program b). Table 3 shows the m/z used for each analyte and the time segment during which, each ion is monitored.

## Results

*Gas chromatographic determination*

Analysis was performed in the SIM mode based on the use of one target and two qualifier ions. Monoterpens were identified according to their retention times, target, and qualifier ions. The quantitation was based on the peak area ratio of the targets to that of internal standard. Table 5 summarizes monoterpenes studied with their target and qualifier ions used in SIM mode in this study.

*Method validation*

*Linearity of the calibration curves*

Calibration curves were constructed for each compound using five different concentration levels. Para-xylene was used as internal standard. For identification of monoterpenes, the retention time and three ions (one for quantitation and two for identification) were used.

The 9 monoterpens showed linearity in SIM mode. Linear calibration curves for all the terpens were obtained with correlation coefficients higher than 0.994. Table 6 shows calibration data (equation and regression coefficient) of all the monoterpens in this study. The calibration curve for α-pinene is presented in Figure 2 as an example.

*Limit of detection and limit of quantification*

The quantification limit (LOQ) was determined by analysis of the samples with known concentrations of analytes and by establishing the minimum level at which the analyte could be quantified with 20% accuracy according to ICH guideline. LOD was calculated as one third of LOQ.

*Precision and accuracy*

The intra-day and inter-day precision were determined by analyzing three replicates of quality control samples at 1/2, 1/4, and 1/8 of original concentration of each monoterpen in the stock sample on the same day and three times on three different days, respectively. The precision was evaluated by the relative standard deviation (RSD) and the acceptable range of RSD was no more than 15% (14). The accuracy was assessed by the methodology recovery. The recovery of the method was calculated by comparing the calculated concentration of QC samples to their actual concentrations. Table 7 presents the recovery and repeatability of 3 concentration levels. The recovery of monoterpens at 3 concentration levels were in the range of 92.4- 120%. The method was proved to be repeatable with RSDs in the range of 80-120% at all spiking levels.

## Results

The quality control of herbal drugs is a big challenge in all around the world. World health organization has issued guidance to validate the herbs that are used for medicinal and therapeutics purposes. 

There is a need to develop a validated system through which the quality of the herbal products can be evaluated and approved in terms of the presence or absence of its chemical constituents as well as quantitative determination of them.

GC-MS is an ideal technique for the separation, identification, and quantification of herbal products containing volatile and semivolatile components such as monoterpenes. Mass detector attached to the GC has an extra advantage through which each separated constituent can be fragmented and the pattern of fragmentation can be compared with the spectra available in the database. 

This capability of mass detector will provide the information of the constituents presented in the herbal product and eventually, all the available constituents can be separated, fragmented, characterized, and quantified simultaneously. 

In the present work, an optimized temperature program was used to separate the 7 monoterpenes as constituents of Rowatinex and the method was fully validated.

Using the temperature program which was developed for separation of chemical constituents of Rowatinex, failed to separate some of the constituents of Rowachol namely menthol, menthone and borneol. Even though the structures of menthol, menthone, and borneol are somewhat different, it seems that the affinity to HP-5 stationary phase is similar for the three compounds. The three compounds co-elute at around 15 min by this temperature program. Different ramp rates which tryingto increase the oven temperature, did not improve the separation. Since we have achieved a good separation for the first five components (p-xylene, α-pinene, β-pinene, camphene and cineol) and we just needed to separate the remaining three compounds (menthol, menthone and borneol), we decided to start the temperature program similar to the program which was used for Rowatinex and after elution of the first five components, we decreased the temperature from 130 °C to 70 °C in order to cause a short-time retention followed by increasing the temperature at 30 °C/min. This strategy gave us an acceptable separation of the remaining three constituents of Rowachol *i.e*. menthol, menthone, and borneol.

## Conclusion

A new, simple and rapid GC assay method has been developed for the simultaneous analysis of 7 monoterpens in certain commercial pharmaceutical dosage forms (Rowatinex and Rowachol).

The method is simple, rapid, accurate, and economical. The designed method has been validated and it showed acceptable linearity, precision, accuracy, and system stability.
